# Characteristic CT Imaging Features of Atraumatic Peritoneal and Subperitoneal Pathologies in Emergency Settings: A Pictorial Review

**DOI:** 10.7759/cureus.95222

**Published:** 2025-10-23

**Authors:** Christopher D Louviere, Jennifer L Wen, Neal M Hall, Ruben G Ortiz, Dheeraj R Gopireddy, Grit A Adler, Renato Abu Hana

**Affiliations:** 1 Department of Radiology, University of Florida College of Medicine – Jacksonville, Jacksonville, USA

**Keywords:** ct imaging, emergency radiology, mesenteric disorders, peritoneal disease, subperitoneal

## Abstract

Severe abdominal pain is a common presentation in emergency departments (ED) and may indicate atraumatic peritoneal or subperitoneal pathology. Imaging is a mainstay in the ED workup for such conditions and is crucial in guiding treatment in time-critical cases. However, the similarities in clinical presentation and imaging features between these pathologies can often make diagnosis more difficult. The purpose of this case series is to describe and compare characteristic CT imaging findings of infectious, inflammatory, cystic, and solid peritoneal and subperitoneal pathologies frequently encountered in the ED. Accurate interpretation of CT imaging features, such as fat stranding, mass effect, nodularity, and fluid collections, is critical for timely diagnosis and treatment of these diverse conditions.

## Introduction

The peritoneum comprises two layers: the parietal peritoneum lining the abdominal wall and the visceral peritoneum covering internal organs. Between these layers lies the peritoneal cavity, while the subperitoneal space connects to the retroperitoneum and contains fat, lymphatics, nerves, and blood vessels [1,2]. Both regions can host a variety of pathologies that often present in emergency settings with severe abdominal pain or nonspecific symptoms [3].

CT is the preferred imaging modality for these scenarios due to its superior resolution and ability to detect subtle differences in fluid, soft tissue, inflammation, and masses [1]. However, the overlapping clinical and imaging presentations of many peritoneal conditions can complicate diagnosis. Here, we summarize key characteristics such as epidemiology and clinical presentation for a multitude of atraumatic peritoneal and subperitoneal conditions. Through a selection of representative images, this series aims to illustrate and compare characteristic CT patterns across 17 conditions, serving as a tool for clinicians to aid in the accurate and prompt diagnosis of peritoneal and subperitoneal disease.

Infectious and inflammatory conditions

Epiploic Appendagitis

Epiploic appendagitis is a benign, self-limiting ischemic/inflammatory condition usually caused by the torsion or venous thrombosis of small, fat-containing pouches on the colon known as epiploic appendages. Epiploic appendages are covered by the visceral peritoneum and are usually 1-2 cm thick and 2-5 cm long (up to 10 cm), with narrow and longer appendages increasing the risk for torsion or venous thrombosis. Other risk factors include a history of obesity and rapid weight loss, as these patients are prone to having large epiploic appendages [3,4]. Although the exact incidence rate is unknown, epiploic appendagitis is estimated to account for 2-7% of cases initially evaluated for suspected appendicitis or diverticulitis [3]. Patients most commonly present with acute, focal, non-migratory, left lower quadrant pain. However, right lower quadrant and hypogastric pain are also possible [3,5]. This presentation can be correlated with the most commonly affected colonic segments, which include the sigmoid colon (approximately 50% of cases), the descending colon (26%), the cecum and ascending colon (22%), and the transverse colon (2%) [3].

Treatment of epiploic appendagitis usually consists of conservative management and nonsteroidal anti-inflammatory drugs (NSAIDs). Despite the benign and self-limiting nature of this condition, its ability to mimic appendicitis and diverticulitis makes appropriate diagnosis crucial to prevent unnecessary hospitalizations, antibiotic use, and surgery [3-5].

Omental Infarction

Omental infarction is known to be a rare, self-limiting, idiopathic, or postoperative focal ischemic necrosis of the greater omentum that classically mimics acute abdomen pathologies such as appendicitis. It is associated with factors such as obesity, hyperperistalsis, rapid body movement, trauma, and prior abdominal surgery [6]. Patients typically present with sudden-onset, right-sided abdominal pain, occasionally accompanied by nausea, vomiting, low-grade fever, or a palpable mass.

Because omental infarction is generally self-limiting, conservative management with NSAIDs and supportive care is preferred, with symptoms resolving in an average of two weeks. Surgery is reserved for patients with complications or concerns for abscess formation [6].

Mesenteric Panniculitis

Mesenteric panniculitis is characterized by inflammation of the mesenteric fat, with or without accompanying fibrosis. If fibrosis predominates, the condition is referred to as sclerosing mesenteritis. The condition is rare, with a reported prevalence of 0.16% to 7.8% on abdominal CT imaging and most commonly occurring in oncologic patients [7,8]. The etiology of mesenteric panniculitis is unclear, with possible causes including prior abdominal surgery or trauma, autoimmune disease, chronic infection, and malignancies [7]. Although up to 40 percent of cases may be asymptomatic and detected incidentally, some patients may present with symptoms such as abdominal pain, bloating, distention, altered bowel habits, nausea, vomiting, anorexia, weight loss, fever, and malaise [7,8]. Additionally, CT findings of mesenteric panniculitis can coexist with additional abdominal malignancies, most commonly lymphomas [7]. Due to this, a thorough evaluation and prompt diagnosis are needed to avoid delays in oncologic care.

Management typically includes observation and periodic imaging for asymptomatic patients, while those with symptoms are generally treated with corticosteroids, tamoxifen, or colchicine. Immunosuppressive or biologic therapies like infliximab have also provided some benefit to patients with longstanding mesenteric panniculitis. Surgery is rarely indicated and is generally limited to cases of bowel obstruction [7,8].

Mesenteric Adenitis

Mesenteric adenitis is a self-limiting inflammatory condition involving the mesenteric lymph nodes. It commonly mimics appendicitis in adults and intussusception in children and causes right lower quadrant abdominal pain, fever, nausea, vomiting, and altered bowel habits. The diagnosis is considered to be primary when findings occur in the absence of an intra-abdominal inflammatory source and secondary when found in the setting of appendicitis, inflammatory bowel disease, systemic inflammatory disorders, or an intra-abdominal infection [9].

Management is typically conservative with hydration and analgesia, as symptoms tend to resolve within two to four weeks [9]. Nonetheless, prompt diagnosis is paramount to prevent unnecessary surgical interventions.

Sclerosing Mesenteritis

Sclerosing mesenteritis is a rare fibrotic variant of mesenteric panniculitis that is also referred to as retractile mesenteritis or liposclerotic mesenteritis. Although the etiology is unclear, reported associations include prior abdominal surgery or trauma, autoimmune diseases such as retroperitoneal fibrosis, Sjögren’s syndrome, or sclerosing cholangitis, intra-abdominal malignancy, particularly lymphoma, chronic infections, and, in some cases, possible IgG4-related disease [10]. Patients may be asymptomatic or present with symptoms such as abdominal pain, weight loss, diarrhea, nausea, vomiting, bloating, constipation, fever, or a palpable abdominal mass.

Asymptomatic patients can be managed conservatively with observation and periodic imaging, while symptomatic cases are often treated with corticosteroids, sometimes in combination with agents such as tamoxifen or colchicine, and immunosuppressive or biologic therapies for refractory disease. Surgical management is reserved for treatment-resistant patients or patients with bowel obstruction [8,10].

Mesenteric Fibro-Fatty Proliferation

Mesenteric fibro-fatty proliferation, or creeping fat, is a pathological expansion of mesenteric adipose tissue encasing diseased bowel segments. It is closely linked to long-standing ileal or ileocolonic Crohn’s disease, transmural inflammation, muscular hypertrophy, fibrosis, and stricture formation. Imaging with CT enterography is used to quantify the Mesenteric Creeping Fat Index, which can predict early postoperative recurrence of Crohn’s after bowel resection [11].

There is no specific therapy for creeping fat, and management focuses on controlling underlying Crohn’s disease activity. Surgical resection of diseased mesentery is under investigation as a strategy to reduce postoperative recurrence risk [11].

Tuberculous Peritonitis

Abdominal tuberculosis (TB) is one of the most common forms of extrapulmonary TB and can involve the peritoneum. Several types of peritoneal TB exist, including the “wet type,” “dry type,” or “fibrotic-fixed” [12]. The wet type is characterized by large amounts of free or loculated ascites, with smooth peritoneal thickening. “Dry type” is characterized by caseous nodules and fibrous adhesions. “Fibrotic-fixed” TB presents with omental mass formation and matting of the bowel loops and mesentery, and may have loculated ascites. Risk factors for peritoneal TB include HIV infection, immunosuppression, or being in contact with TB through migration or travel. Patients can present with lymphadenopathy, ascites, abdominal distention, altered bowel habits, and nonspecific symptoms such as abdominal pain or fever [12,13].

The diagnosis of tuberculous peritonitis requires differentiation from other pathologies such as disseminated peritoneal malignancy, non-tuberculous peritonitis, and mesothelioma to provide patients with the proper management [13].

Intraperitoneal Abscess

Intraperitoneal abscess is defined as a collection of pus within the peritoneal cavity and most commonly arises as a postoperative complication, following abdominal trauma, or by direct spread from infected organs. Clinical presentation varies and includes fever, abdominal pain, localized tenderness, anorexia, nausea, and malaise [14].

Management of intraperitoneal abscesses includes antibiotic therapy with source control, typically through image-guided (CT or ultrasound) percutaneous drainage. Prompt intervention reduces morbidity and hospital stay. Surgical incision and drainage can be used on a case-by-case basis [14].

Gossypiboma

Gossypibomas, or textilomas, are the result of foreign materials, surgical gauze, swabs, or sponges, being improperly left in the body post-surgery. Risk factors include emergency surgery, unplanned procedural changes, high body mass index, excessive blood loss, prolonged or complex operations with multiple teams, incorrect sponge or instrument counts, and the female sex due to higher rates of gynecologic surgery. The clinical presentation of gossypibomas is variable depending on the localization of retained foreign materials, but can be associated with a palpable mass and fever [15]. Definitive management is the surgical removal of the entrapped objects.

Cystic masses

Pseudomyxoma Peritonei (PMP)

PMP is a rare form of cancer characterized by mucin-secreting tumor cells that affect the peritoneal lining of the abdominal cavity. It is divided into low-grade and high-grade based on histopathology [16]. Clinically, PMP lacks distinct signs and symptoms; however, nonspecific findings such as abdominal distention and bowel obstruction can arise as the disease progresses. CT findings such as massive ascites and peritoneal solid nodules serve as independent predictors of high-grade pathology. Treatment of PMP includes cytoreductive surgery combined with hyperthermic intraperitoneal chemotherapy [16].

Mesenteric Cysts

Mesenteric cysts are a rare group of heterogeneous intra-abdominal cystic lesions that originate in the mesentery or omentum. They can develop anywhere within the mesentery of the gastrointestinal tract, from the duodenum to the rectum, and can extend from the base of the mesentery into the retroperitoneum. While the etiology is currently unknown, some proposed mechanisms include developmental abnormalities, trauma, prior surgery, and lymphatic drainage abnormalities. They are often asymptomatic and seen incidentally on imaging. When present, symptoms include chronic abdominal pain, distention, nausea, vomiting, diarrhea, or the presence of a palpable abdominal mass. In the emergent setting, patients can present with an acute abdomen secondary to mesenteric cyst complications such as intestinal obstruction, volvulus, hemorrhage, rupture, infection, or peritonitis [17].

The treatment of choice for mesenteric cysts is surgical excision, especially when solid components are present, as they have an increased risk for malignant transformation. When mesenteric vessels are involved, partial resection of the affected bowel may be necessary. Less invasive treatment options, such as percutaneous drainage, can also be used, but these carry an increased risk for recurrence [17].

Cerebrospinal Fluid (CSF) Pseudocysts

Peritoneal CSF pseudocysts are defined as CSF-filled cysts that are contained within a non-epithelial lining. They occur as rare complications of ventriculoperitoneal (VP) shunt placement and are more commonly seen in the pediatric population. The formation of CSF pseudocysts has been associated with interrupted peritoneal absorption of CSF, shunt blockage, or abdominal adhesions. Patients can present with nonspecific abdominal complaints, including distention, diffuse tenderness, nausea, vomiting, or bowel obstruction in larger cysts [18].

Currently, treatment options include surgical excision of the cyst with repositioning of the shunt, CT-guided aspiration, paracentesis, and targeted antibiotic therapy after a CSF sample has been obtained [18]. Ultimately, the optimal therapeutic intervention will be dependent on the patient's status and clinical presentation.

Solid masses

Sarcomas

Peritoneal and retroperitoneal sarcomas are both rare malignant tumors. Peritoneal sarcomatosis typically appears as smooth, well-circumscribed, multinodular masses, whereas retroperitoneal sarcomas present as large, heterogeneous masses. Gastrointestinal stromal tumors (GISTs) are the most common intraperitoneal sarcomas, while liposarcomas and leiomyosarcomas are the first and second most common retroperitoneal sarcomas [19]. Patients usually present with nonspecific symptoms, such as fever, weight loss, or abdominal pain. As a result, diagnosis is commonly made when the lesions have progressed significantly in size and present with areas of necrosis or hemorrhage [19,20].

Management will depend on the subtype and stage at diagnosis; surgery is generally the mainstay of therapy for soft-tissue sarcomas [19].

Gastrointestinal Stromal Tumors (GISTs)

GISTs are rare submucosal tumors that most often arise from the stomach or small intestine and account for approximately 1-2% of all gastrointestinal cancers. Other common locations for GISTs include the colon, rectum, and rarely the esophagus. GISTs have the potential to undergo malignant transformation in around 10-30% of cases, with extragastric GISTs having an even higher potential for malignancy. Symptoms can range from asymptomatic to GI bleeds to perforation and intraperitoneal bleeding [21].

The gold standard management of GISTs consists of complete surgical resection if possible. Adjuvant chemotherapy with imatinib may also be used in high-risk patients. After receiving treatment, high-risk patients may need serial abdominal CT imaging every three months for five years for continuous monitoring [21].

Inflammatory Myofibroblastic Tumors (IMTs)

IMTs are rare tumors made up of myofibroblastic and fibroblastic spindle cells as well as inflammatory cells, with lesions found in the mesentery, omentum, or retroperitoneal space. They are more common in children and young adults with reported risk factors including previous trauma, abdominal surgery, or mutations in the anaplastic lymphoma kinase (ALK) gene [22]. Clinical presentation is dependent on the site of origin, but most patients present with non-specific symptoms, including fever, malaise, weight loss, abdominal mass, and gastrointestinal symptoms. On CT imaging, IMTs may have central necrosis but usually present without calcifications or enlarged lymph nodes [22].

Typically, IMTs are associated with a good prognosis and low recurrence. For localized, less aggressive lesions, management involves surgical resection. In cases where the tumor is unresectable or multifocal disease is present, management may require chemotherapy agents such as Adriamycin or ifosfamide [22].

Peritoneal Carcinomatosis (PC)

PC is defined as the spread of any non-peritoneal primary carcinoma to the peritoneum [23]. It is the most common type of secondary malignancy in the peritoneum and frequently originates from ovarian, breast, gastric, or colorectal cancer. These are much more common in adult patients and the female population [20,24]. In terms of CT imaging, while small, discrete nodules may be present, they are often poorly defined and possess irregular borders [24]. Mesenteric stranding and soft tissue plaques may also be visualized.

Treatment focuses on managing primary tumors with systemic chemotherapy. Patients with primary colorectal, appendiceal, or ovarian carcinomas may undergo cytoreductive surgery or hyperthermic intraperitoneal chemotherapy [20,23].

Peritoneal Lymphomas (PL)

PL occurs when there is intraperitoneal spread of a lymphoma, most frequently seen in non-Hodgkin lymphoma (NHL). Among the NHL subtypes, diffuse large B-cell lymphoma is the most commonly associated with peritoneal involvement [20,24].

Primary treatment is non-surgical with systemic chemotherapy. Regarding outcomes, although patients with aggressive NHL have a poorer short-term outlook, more than 70% can be cured with prompt initiation of intensive combination chemotherapy. By contrast, those with a more indolent course of NHL often relapse despite initially having a quick response to pharmacotherapy [25]. Fluorodeoxyglucose (FDG)-PET/CT is important in staging, treatment planning, and monitoring treatment response [20].

## Materials and methods

To elucidate the characteristic imaging patterns of atraumatic peritoneal and subperitoneal mass pathologies, we retrospectively reviewed clinical cases from the University of Florida Health - Jacksonville network between 2015 and 2025 using the mPower image search engine. Inclusion criteria included adults aged ≥ 18 years, with initial search terms limited to CT imaging with “peritoneal,” “mass,” “tumor,” and “subperitoneal” in the impressions section of the results. Exclusion criteria included non-CT images, outpatient examinations, images without thin sections, and cases without overt presentation. This search generated over 115 results, which were then subfiltered according to each particular entity. Representative cases were then selected based on diagnostic clarity and completeness of relevant imaging features, as determined by two board-certified radiologists. Permission to use the images was obtained from each patient whose pathology was selected for inclusion. Data points from each case, including demographics (age and sex) and imaging findings and results, were obtained for each selected case and compiled. Images were then deidentified and stored on secure network computers accessed only by study personnel. An image series was subsequently compiled regarding the salient features described in the reports.

## Results

Images were collected for 17 of the peritoneal and subperitoneal mass pathologies we describe. All patients identified as “White individuals” and “male” in their self-reported demographics, though this was not a feature for which we controlled or selected (Table 1). No patient presented with more than one of the discussed pathologies.

**Table 1:** Patient demographics.

Case Type	Race	Sex	Age	Notable Features
Epiploic appendagitis	White	Male	48	Patient presented with sharp left lower quadrant abdominal pain.
Omental infarction	White	Male	81	Patient presented with acute abdominal pain.
Mesenteric panniculitis	White	Male	59	Patient presented with persistent, dull abdominal pain.
Mesenteric adenitis	White	Male	49	Patient presented for suspected appendicitis.
Sclerosing mesenteritis	White	Male	56	Patient presented with diarrhea and weight loss.
Mesenteric fibro-fatty proliferation	White	Male	55	Patient presented with longstanding Crohn’s disease.
Tuberculous peritonitis	White	Male	39	Patient presented with generalized nonspecific symptoms.
Intraperitoneal abscess	White	Male	40	Patient presented with acute abdominal pain.
Gossypiboma	White	Male	51	Patient presented with nonspecific abdominal pain.
Pseudomyxoma peritonei	White	Male	62	Patient presented with chronic abdominal pain.
Mesenteric cyst	White	Male	55	Patient presented with chronic abdominal pain.
Cerebrospinal fluid pseudocyst	White	Male	58	Patient presented with nausea, vomiting, and abdominal tenderness.
Sarcoma	White	Male	65	Patient initially presented with nonspecific symptoms and weight loss.
Gastrointestinal stromal tumor	White	Male	66	Patient presented with anorectal bleeding.
Inflammatory myofibroblastic tumor	White	Male	37	Patient initially presented with nonspecific symptoms.
Peritoneal carcinomatosis	White	Male	64	Patient presented for treatment of colorectal cancer.
Peritoneal lymphoma	White	Male	51	Patient presented for treatment of non-Hodgkin lymphoma.

Infectious and inflammatory conditions

Epiploic Appendagitis

**Figure 1:**
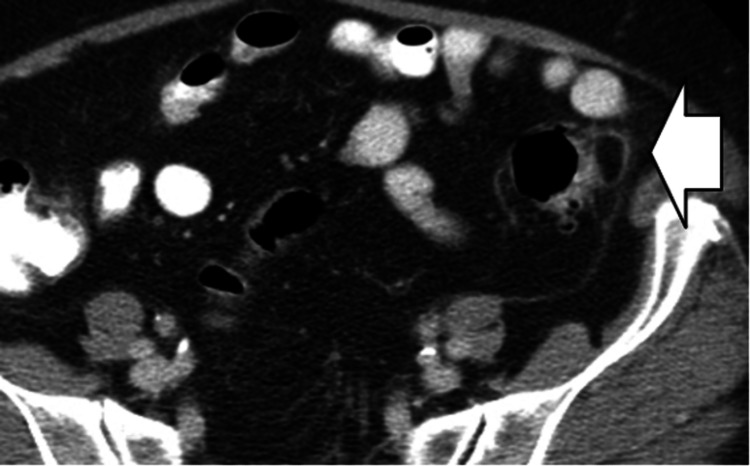
Epiploic appendagitis. CT image shows soft tissue stranding around the epiploic appendage (white arrow) extending off the sigmoid colon.

CT imaging of epiploic appendagitis demonstrates an ovoid fat-density lesion near the colon (Figure 1), with surrounding fat stranding and thickening of the epiploic appendage’s visceral peritoneal lining, deemed the “hyperattenuating ring sign”.

Omental Infarction

**Figure 2:**
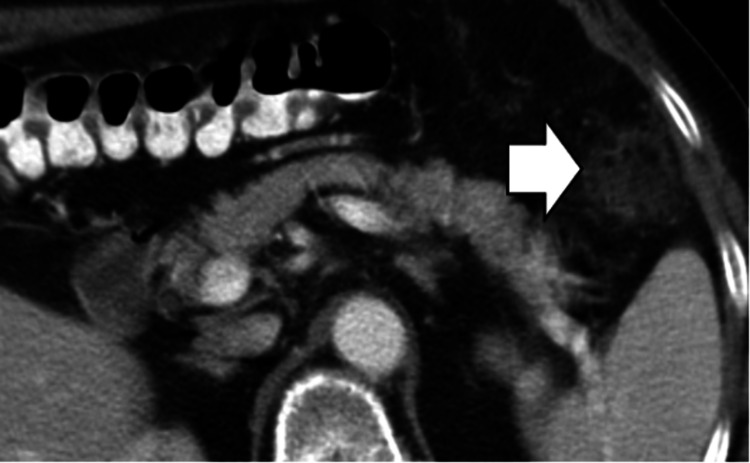
Omental infarct. CT image shows localized soft tissue stranding in the left upper quadrant omentum (white arrow).

CT imaging findings of an omental infarction include heterogeneous fat-density lesions with surrounding fat stranding or mildly hyperdense rims (Figure 2).

Mesenteric Panniculitis

**Figure 3:**
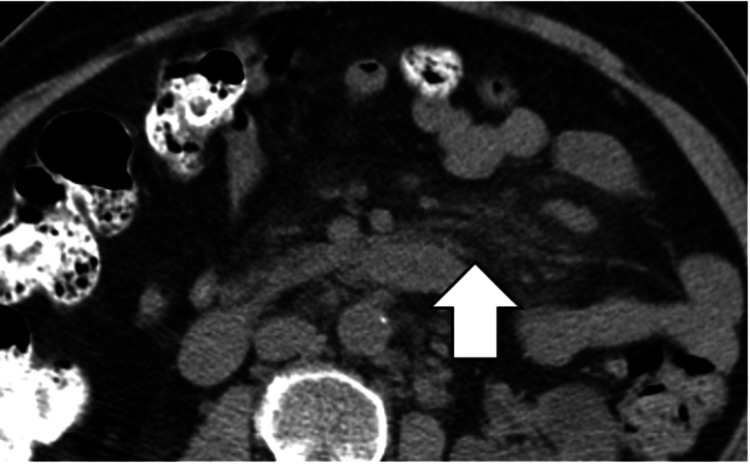
Mesenteric panniculitis. The CT image shows a misty appearance of the mesentery (white arrow) adjacent to the jejunum with small and prominent lymph nodes and a mild hyperattenuating pseudocapsule.

CT imaging of mesenteric panniculitis shows soft-tissue attenuation of mesenteric fat (Figure 3), with a pseudocapsule or fat halo sign.

Mesenteric Adenitis

**Figure 4:**
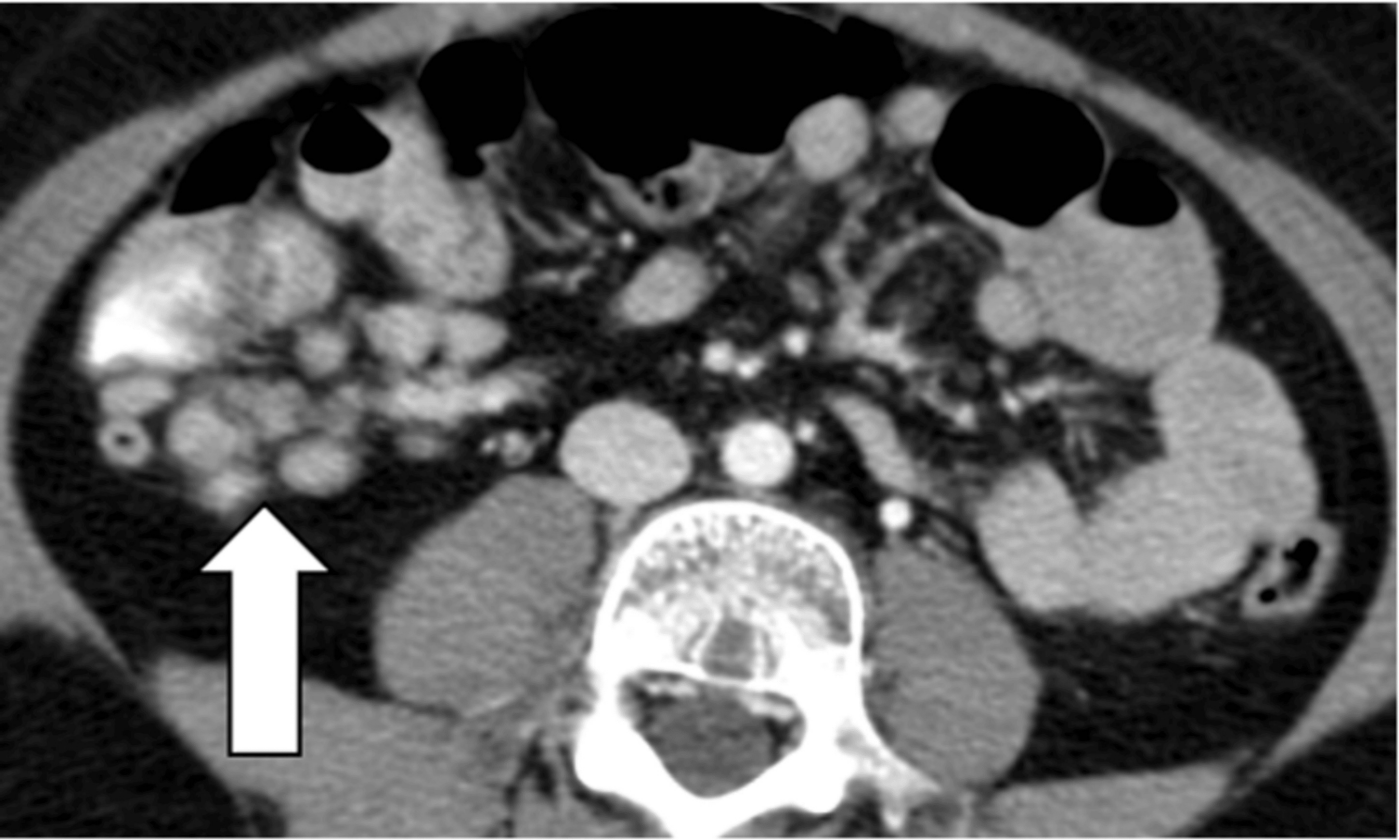
Mesenteric adenitis. CT image shows multiple ileocecal nodes greater than 5 mm in diameter (white arrow), adjacent to a normal, air-filled appendix.

On CT imaging, a cluster of at least three mesenteric lymph nodes in the right lower quadrant, each measuring ≥ 5 mm, and a normal appendix support the diagnosis of primary mesenteric adenitis (Figure 4).

Sclerosing Mesenteritis

**Figure 5:**
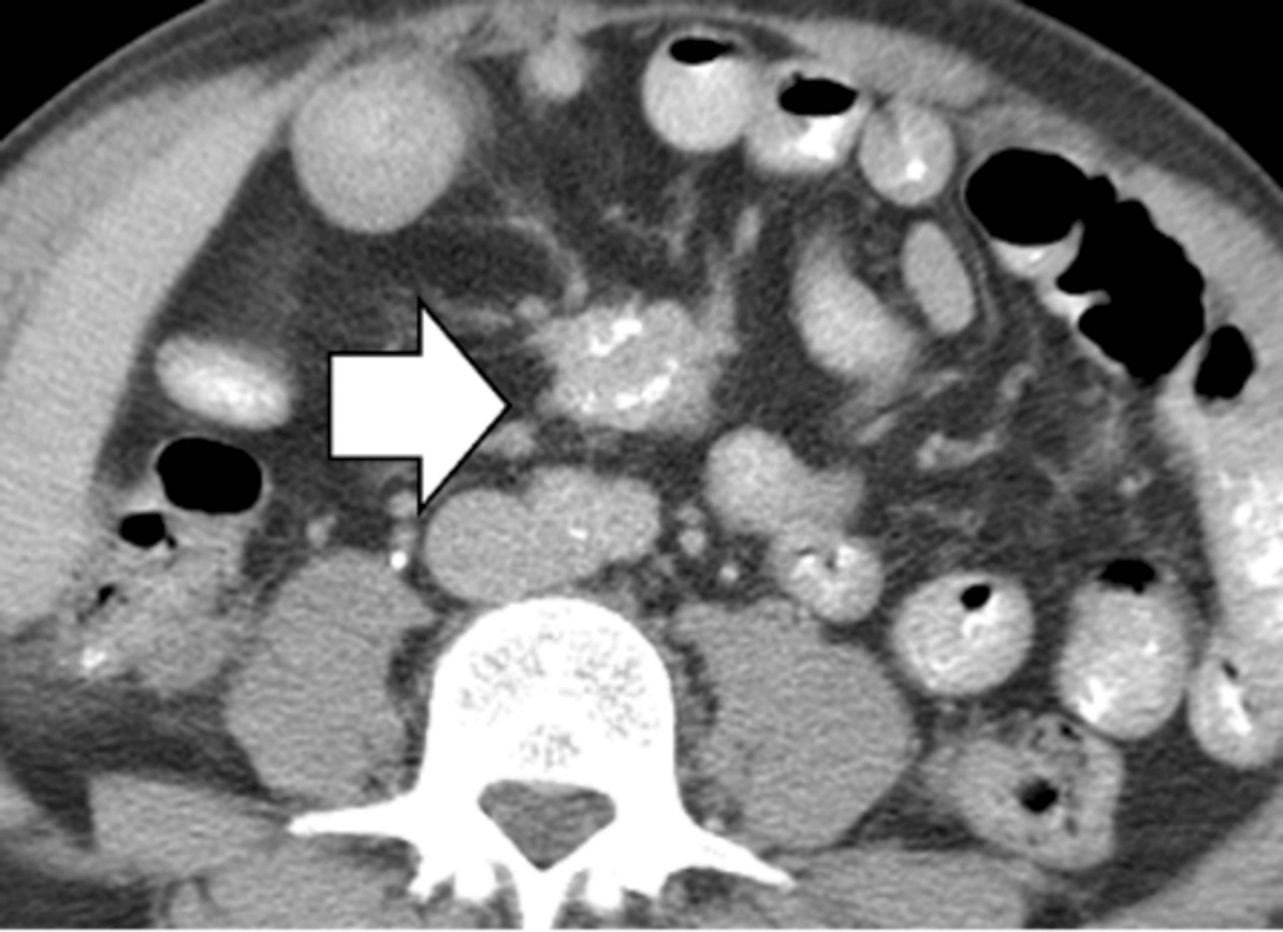
Sclerosing mesenteritis. CT image shows an ill-defined, infiltrating soft-tissue mass (white arrow) in the root of the mesentery with associated calcification and desmoplastic reaction.

The appearance of sclerosing mesenteritis on CT imaging closely parallels that of mesenteric panniculitis, with a mesenteric fatty lesion that exhibits higher attenuation, exerts a mass effect on surrounding structures, and may contain soft tissue lymph nodes (Figure 5).

Mesenteric Fibro-Fatty Proliferation

**Figure 6:**
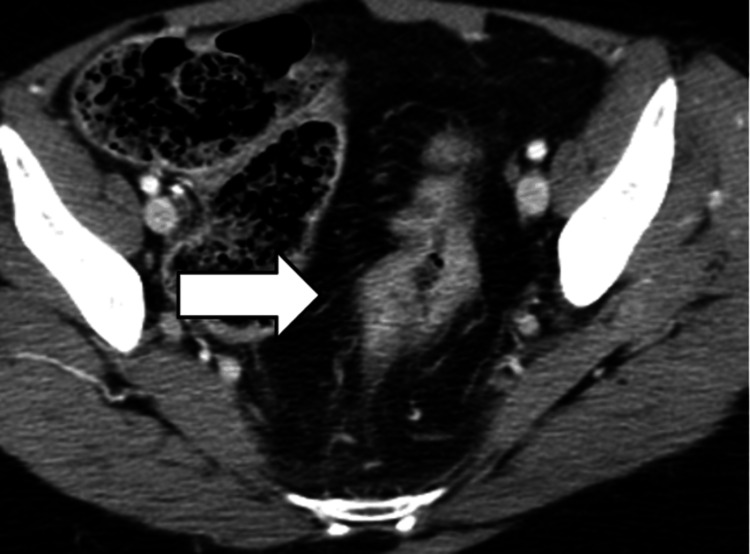
Mesenteric fibro-fatty proliferation. CT image shows mesenteric fibro-fatty proliferation (creeping fat) of the sigmoid mesentery in a patient with Crohn's disease (white arrow).

On CT imaging, mesenteric fibro-fatty proliferation is characterized by fat hyperplasia and stranding around the bowel (Figure 6).

Tuberculous Peritonitis

**Figure 7:**
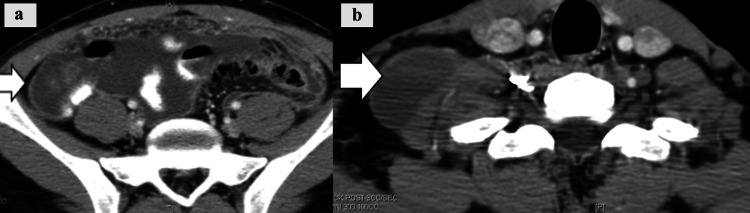
Tuberculous peritonitis. a: CT image shows thickening of omentum (white arrow) with ascites (wet type of TB); b: CT image of the neck shows right supraclavicular lymphadenopathy (white arrow) secondary to TB in the same patient.

In tuberculous peritonitis, CT imaging shows omental thickening and ascites, as well as lymphadenopathy associated with systemic disease (Figure 7).

Intraperitoneal Abscess

**Figure 8:**
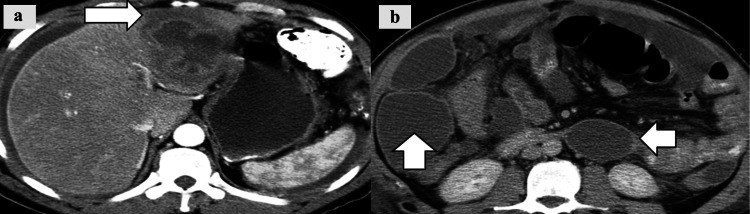
Intraperitoneal abscess. a: CT image shows hepatic abscess (white arrow); b: CT image shows multiple intraperitoneal fluid collections (white arrows) secondary to liver abscess rupture.

The intraperitoneal abscesses appear as abnormal fluid collections with rim enhancement and potential air-fluid levels (Figure 8).

Gossypiboma

**Figure 9:**
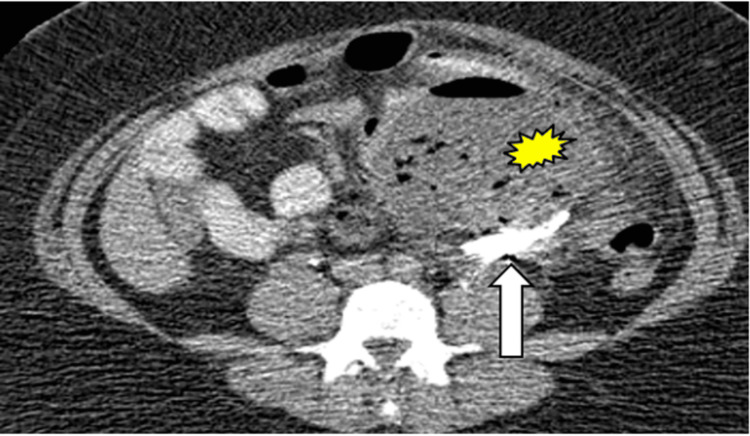
Gossypiboma. CT image shows a large intraperitoneal abscess (yellow burst) with a retained foreign body: a sponge (white arrow).

On CT imaging, gossypibomas have a spongiform/whorled internal pattern, sometimes with trapped gas bubbles or a calcified appearance, and can mimic abdominal tumors or abscesses (Figure 9).

Cystic masses

Pseudomyxoma Peritonei (PMP)

**Figure 10:**
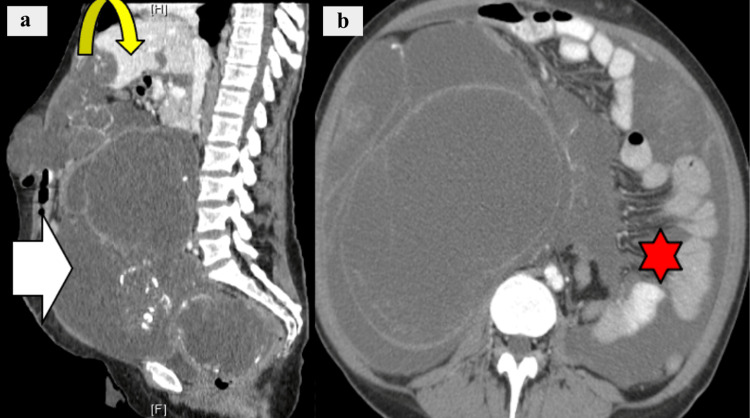
Pseudomyxoma peritonei. a: diffuse peritoneal fluid collections (white arrow) with septations and rim calcifications, and scalloping of the liver margins is seen (yellow arrow); b: resulting displacement of intestines laterally (red star).

On CT imaging, cystic masses and copious mucinous ascites that can cause hepatic scalloping are distinguishing features of PMP (Figure 10). Other findings include omental caking, curvilinear calcifications, and bowel displacement due to mass effect.

Mesenteric Cysts

**Figure 11:**
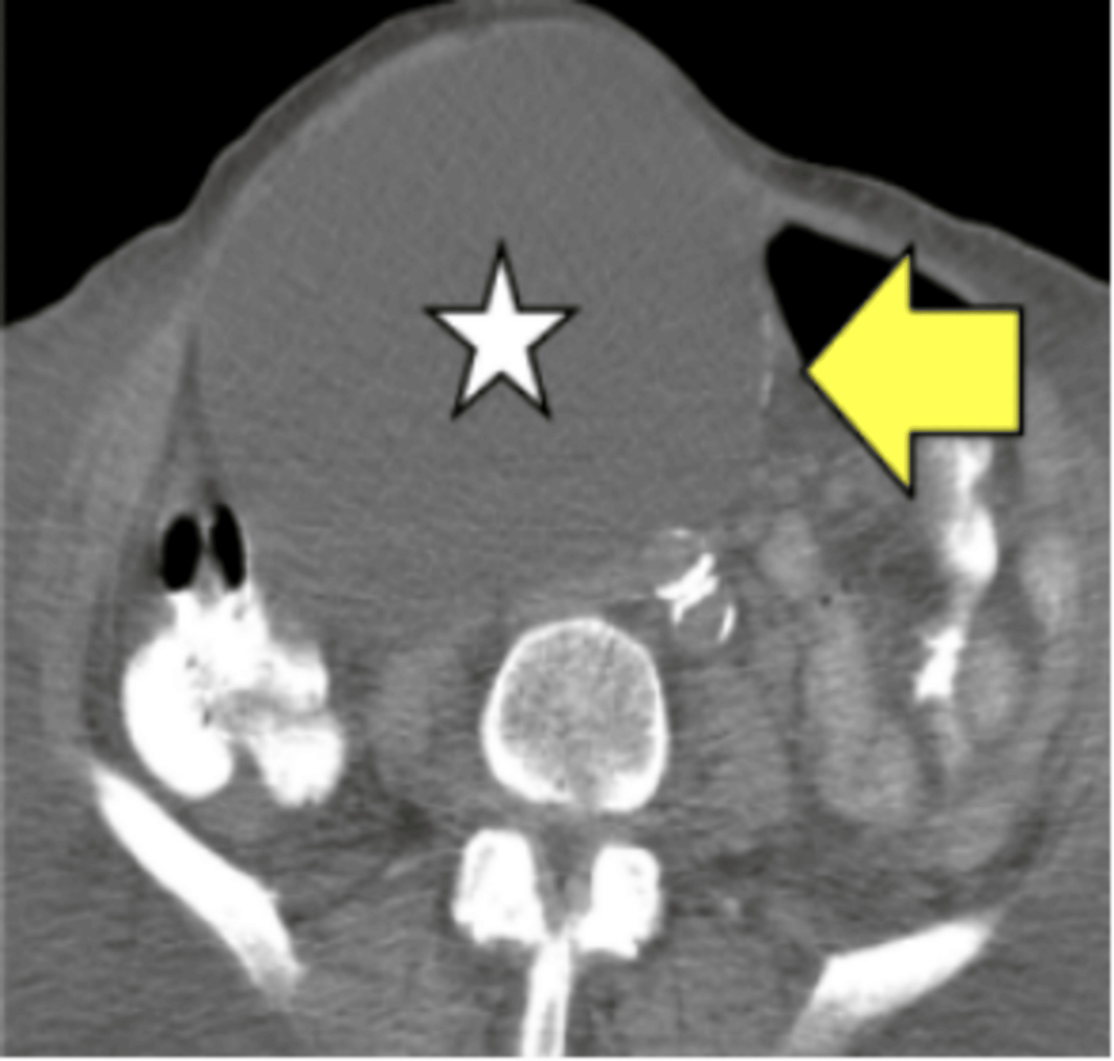
Mesenteric cyst. CT image shows a mesenteric cyst (white star) with a rim of calcification (yellow arrow).

On imaging, the mesenteric cyst is visualized as a fluid-filled, well-circumscribed mass that has calcifications (Figure 11).

Cerebrospinal Fluid (CSF) Pseudocysts

**Figure 12:**
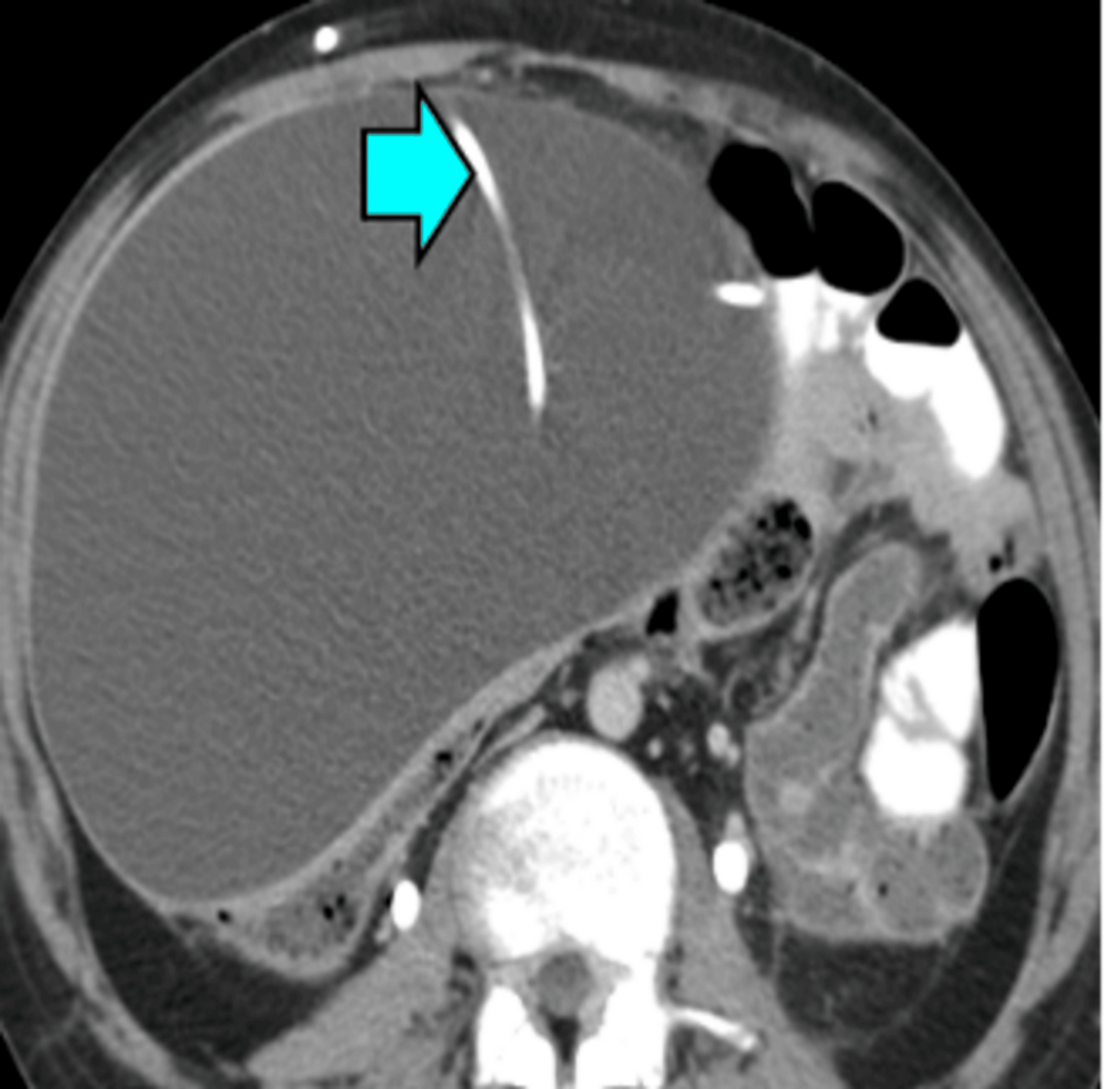
CSF pseudocyst. Large fluid collection associated with a shunt (blue arrow) in the peritoneal cavity on CT imaging.

On CT imaging, the CSF pseudocyst presents as a thin-walled, cystic mass at the tip of a VP shunt catheter (Figure 12).

Solid masses

Sarcomas

**Figure 13:**
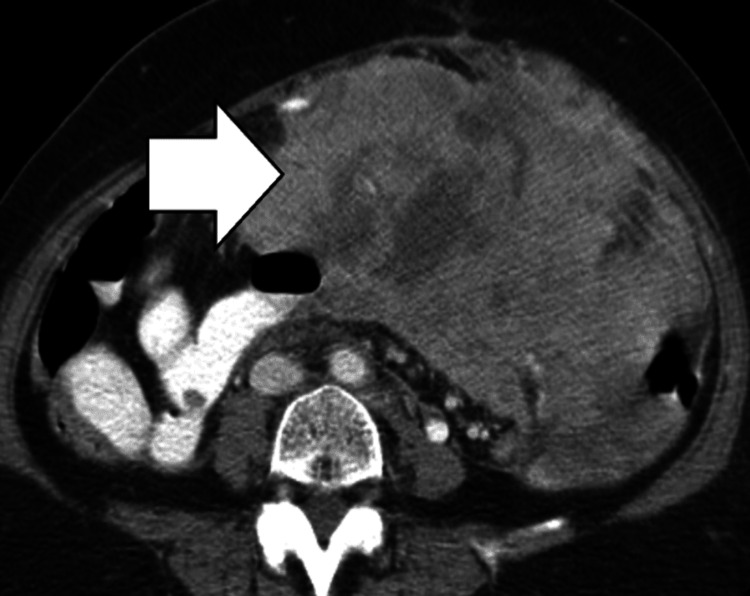
Peritoneal sarcoma. CT image shows a large heterogeneously enhancing intraperitoneal mass arising from the peritoneum (white arrow).

On imaging, the nodules in peritoneal sarcomatosis present as large, heterogeneous, and hypervascular, with areas of hemorrhage, necrosis, or myxoid change (Figure 13).

Gastrointestinal Stromal Tumors (GISTs)

**Figure 14:**
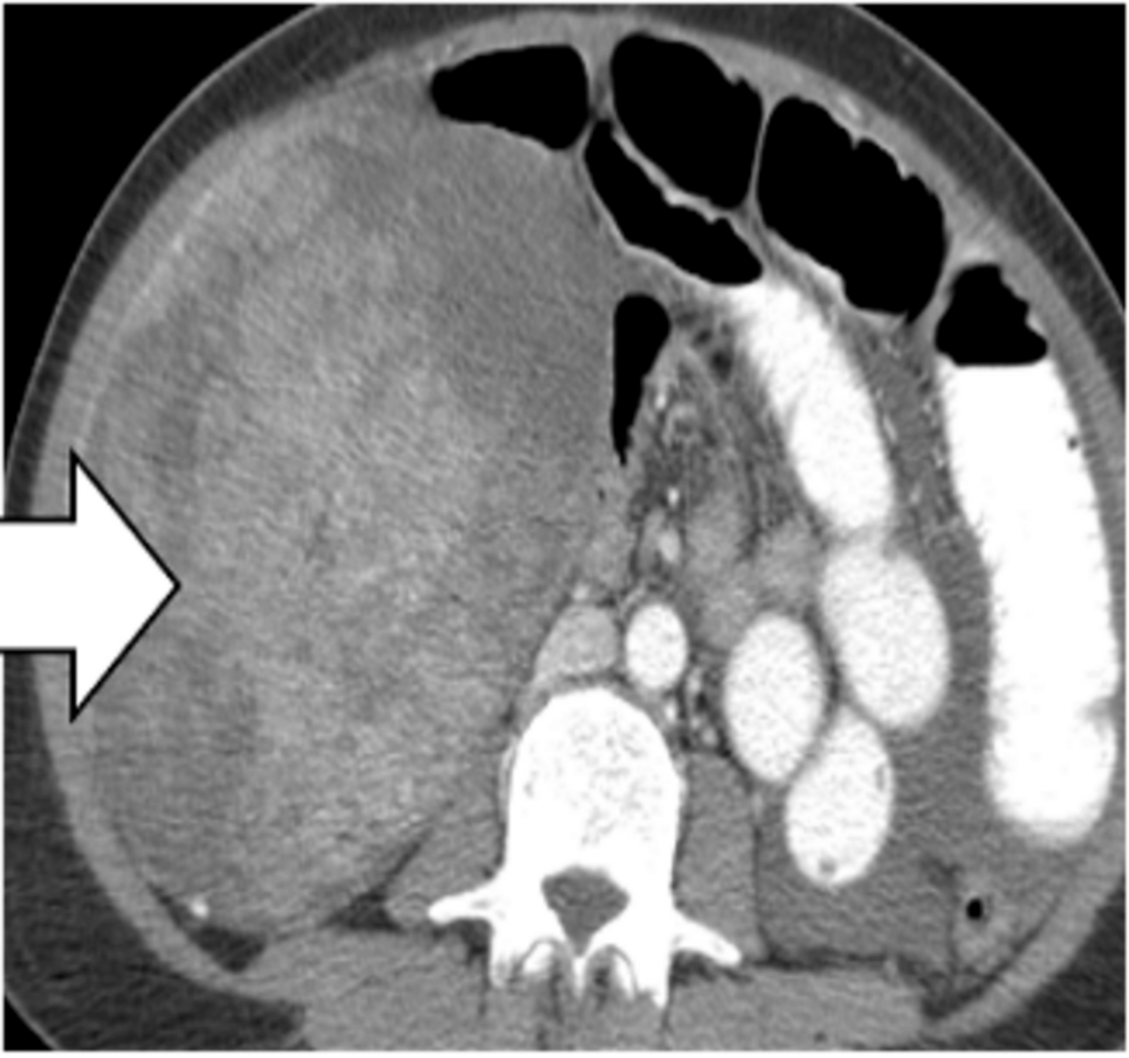
Gastrointestinal stromal tumor (GIST). Large heterogeneous mass in the right lower quadrant (white arrow) of the intraperitoneal cavity in a patient with metastatic GIST.

GISTs appear on CT imaging as masses with heterogeneous enhancement (Figure 14) and potential central necrosis or hemorrhage.

Inflammatory Myofibroblastic Tumors (IMTs)

**Figure 15:**
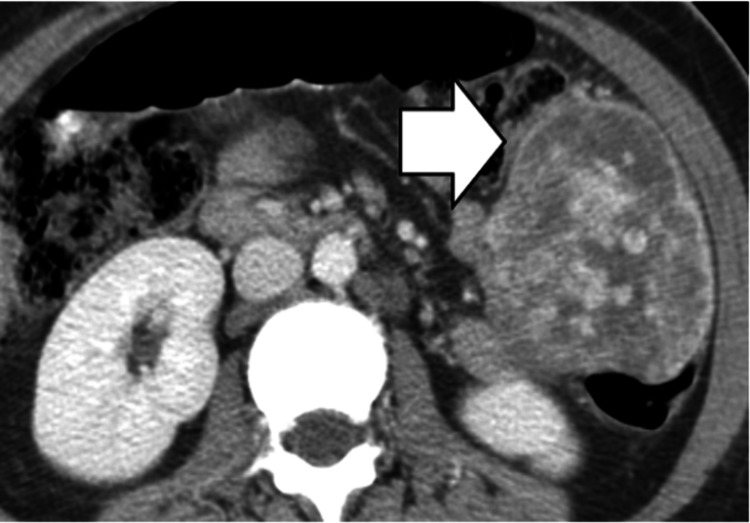
Inflammatory myofibroblastic tumor (IMT). Myofibroblastic tumor presenting as a heterogeneously enhancing mass arising from the omentum (white arrow).

IMTs appear as hypervascular, heterogeneously enhancing masses (Figure 15), with tumor margins ranging from well-defined to infiltrating.

Peritoneal Carcinomatosis (PC)

**Figure 16:**
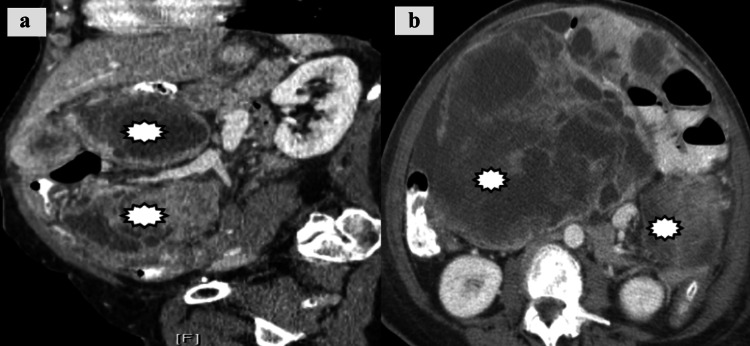
Peritoneal carcinomatosis. Heterogeneous necrotic peritoneal masses (white bursts) secondary to metastatic endometrial carcinoma seen on (a) sagittal and (b) axial on CT imaging.

On CT imaging, PC is characterized by omental caking, free or loculated ascites, and peritoneal thickening/enhancement (Figure 16).

Peritoneal Lymphomas (PL)

**Figure 17:**
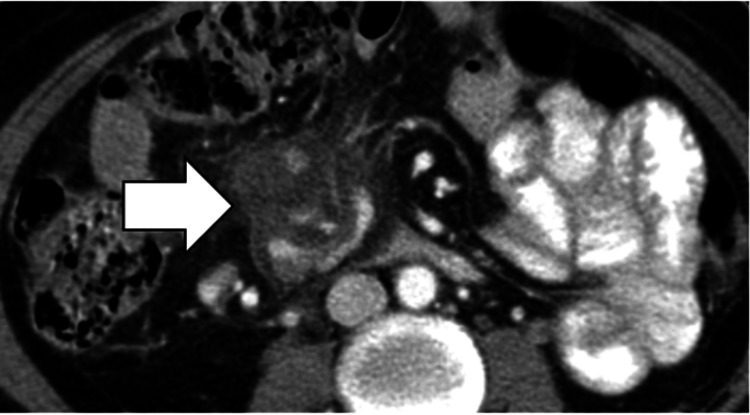
Lymphoma. CT shows a soft tissue mass at the mesenteric root, secondary to Burkitt’s lymphoma (white arrow).

PL presents with ascites, more homogenous omental thickening, and infiltrating mesenteric masses on CT imaging (Figure 17).

## Discussion

Infectious and inflammatory conditions

In the emergency setting, contrast-enhanced CT is the baseline study for suspected infectious or inflammatory disease of the peritoneum and mesentery. Inflammation causes the normally low-attenuation mesenteric fat (approximately -100 to -40 Hounsfield Units (HUs)) to appear as a “misty” mesentery; the fat appears hazy due to edema and stranding [8,26]. Fluid characterization can also assist with triage, as simple serous fluids measure similarly to water (<10 HU), proteinaceous or infected collections trend higher, and blood typically measures ~30 to 45 HU [27,28]. Because attenuation can be influenced by partial volume, image timing, or adjacent hardware, HU measurement should not be used alone for interpretation. Morphology, enhancement features, and clinical status should be considered and weighed appropriately when making a diagnosis.

Cystic masses

When encountering cystic lesions commonly found in the peritoneum and mesentery on CT imaging, evaluating properties such as attenuation and enhancement of walls, septa, or nodules should be prioritized. For example, on unenhanced CT, simple cysts measure near water (0-20 HU), and on contrast-enhanced CT, they show no measurable enhancement. On the other hand, higher attenuation could reflect protein or blood components, while lower attenuation with a fat-fluid level suggests chylous or fat-containing fluid [29].

Solid masses

For solid peritoneal and subperitoneal lesions, the first question should be whether the lesion enhances. Since solid masses can measure around 30-100 HUs, which overlaps with other pathologies, enhancement is key for appropriate differentiation. A rise of more than 20 HU post-contrast CT supports enhancement and thus helps to separate solid tumors from simple cysts or necrotic fluid [30]. Once a lesion’s enhancement status has been determined, focus should be shifted towards the evaluation of attenuation and morphology to further guide differential diagnoses.

Synthesis of imaging features

The peritoneal and subperitoneal spaces can harbor a range of pathologies, from benign and self-limiting to malignant. Patients with these pathologies can present with nonspecific or overlapping symptoms, with severe abdominal pain being a common concern. Thus, the ability to recognize distinguishing features of each pathology type on CT imaging is key to facilitating prompt detection and accurate diagnosis. Here, we compare the imaging findings of this case series to those described in other studies to highlight notable patterns across pathology types.

As seen in this case series and in the wider literature, infectious and/or inflammatory conditions are often characterized by fat stranding. The presence of specific imaging features, such as the “hyperattenuating ring sign” for epiploic appendagitis or a pseudocapsule in mesenteric panniculitis, can be utilized to discern these disorders from each other [3,8,10]. One way to differentiate omental infarction from other acute abdomen pathologies such as appendicitis, diverticulitis, and epiploic appendagitis in CT imaging is the absence of other associated abnormalities in the bowel [6]. While gossypibomas can mimic abdominal tumors or abscesses with their enhancement patterns or the presence of air bubbles, radiopaque markers are important to look for as a sign of a retained foreign body [15].

HUs and enhancement post-contrast can be used to identify cystic versus solid masses on CT imaging. In general, cystic masses may appear as fluid-filled and well-circumscribed, whereas solid masses may possess more irregularity in borders, with potential areas of hemorrhage, necrosis, or cavitation [19-21]. Neoplastic processes are often associated with omental caking and nodularity. Compared with PC, ascites and lymph node involvement are less common in peritoneal sarcomatosis [19,20]. PL can often resemble PC in terms of its omental involvement, peritoneal thickening, and ascites. However, PL presents with more homogeneous omental thickening. Diffuse lymphadenopathy, splenomegaly, and infiltrating mesenteric masses are also key features that distinguish PL from other disease patterns [20,24]. The absence of calcifications in IMTs serves as a point of contrast to discriminate it from other malignancies, such as GISTs [22].

Limitations

Though each image clearly highlights the pathology in question, we were unable to curate an image series to describe the defining characteristics of each type of mass. Image series would allow for better direct comparison of slight variations in pathology appearance. We were limited by the results of our mPower searches as many of these pathologies are infrequent, and the database was limited in its duration of time. The demographically homogenous sample, descriptive nature of analysis, and constriction to cases at a single center may also limit generalizability, so these cases should be viewed as illustrative for key CT patterns seen in peritoneal and subperitoneal disease.

## Conclusions

A wide spectrum of benign and malignant conditions can involve the peritoneal and subperitoneal spaces. These pathologies can present diagnostic challenges due to overlapping clinical signs and symptoms, most notably acute abdominal pain. However, distinctive CT findings, including fat stranding, mass effect, fluid characteristics, and patterns of enhancement, can guide clinicians toward accurate diagnosis. For example, fat stranding often suggests inflammatory processes (e.g., epiploic appendagitis, panniculitis), whereas omental caking and nodularity point toward malignancy (e.g., carcinomatosis, PMP). Predominantly cystic versus solid morphology, in conjunction with HUs and the presence or absence of enhancement, helps distinguish benign cysts from neoplasms. Associated features such as lymphadenopathy or splenomegaly are also important distinguishing factors and may suggest systemic diseases such as lymphoma or TB. Ultimately, prompt recognition of these imaging characteristics is essential for delivering timely, appropriate care in the emergency setting.
